# Expression of calmodulin-related genes in lead-exposed mice

**DOI:** 10.1515/intox-2015-0024

**Published:** 2015-12

**Authors:** Sun Li, Xiao-Lin Liu, Xie-Lai Zhou, Su-Jun Jiang, Hong Yuan

**Affiliations:** Medical School, Hangzhou Normal University, No16, Xue Lin street, Xia Sha, Hangzhou, 310036, Zhejiang, China

**Keywords:** calmodulin, microarray, gene expression, lead, real time PCR

## Abstract

The toxic metal lead is a widespread environmental polutant that can adversely affect human health. However, the underlying mechanisms of lead-induced toxicity are still largely unknown. The mechanism of lead toxicity was presumed to involve cross reaction between Pb^2+^ and Ca^2+^ with calmodulin dependent systems. The aim of the present study was thus to identify differential expression of calmodulin-related genes in the spleen of lead-exposed mice. We performed microarray analysis to identify differentially expressed genes. RNAs from spleen tissue of lead exposed animals (n=6) and controls (n=6) were converted to labeled cRNA and hybridized to Illumina mouse WG-6_v2_Bead Chip. Expression profiles were analyzed using Illumina BeadStudio Application. Real-time RT-PCR was conducted to validate the microarray data. By microarray analysis 5 calmodulin-related genes (MAP2K6, CAMKK2, CXCR4, PHKA2, MYLK) were found to be differently expressed in lead exposed compared with control mice (*p*<0.05). The results of Real-time RT-PCR showed that MAP2K6 and CAMKK2 were up-regulated and CXCR4 was down-regulated in lead exposure, but there were no significant differences in PHKA2 and MYLK expression between the lead exposed and control group. These results show that lead exposure produced significant changes in expression of a variety of genes in the spleen and can affect calmodulin-related gene expression.

## Introduction

The toxic metal lead (Pb) is a widespread environmental contaminant that can adversely affect human health. Lead is known to exert toxic effects on various target organs, mainly on the central nervous, digestive, hematopoietic, renal and immune systems. Lead has been evaluated extensively in human beings and animal studies with regard to its effects on the immune system (Mishra *et al.,* 2003; Bussolaro *et al.,* 2008). There is growing evidence that lead can directly alter cellular physiology at multiple levels, including interference with ion channels and activation of second messengers, particularly calcium-dependent messengers, which ultimately affect transcription factors and gene expression (Hossain *et al.,* 2000; Cui *et al.,* 2005). In human beings, lead-associated changes were reported for T-lymphocyte subpopulations and plasma cytokines (García-Lestón *et al.,* 2012). Lead was shown to target both interleukin-2-dependent proliferation and T cells (Jorissen *et al.,* 2013). The mechanism of lead toxicity was presumed to involve cross reaction between Pb^2+^ and Ca^2+^ with calmodulin dependent systems (Kirberger *et al.,* 2013). Intracellularly, lead replaces calcium as a second messenger, binding with calmodulin more readily than calcium, inducing alteration in protein conformation. This altered conformation leads protein kinases to phosphorate and activate substrate molecules, thus altering various cellular processes (Kern *et al.,* 2000; Wang *et al.,* 2006; Toscano *et al.,* 2005). In an effort to better understand the effect of lead exposure on calmodulin-related gene expression, we performed microarray analysis to identify differential expression of calmodulin-related genes in lead-exposed mice.

## Materials and methods

### Experimental animals

Balb/c mice (10-day-old) were purchased from the Animal Center, Hangzhou Normal University. They were divided randomly into two groups. The model of lead exposure was established by drinking 0.075% lead acetate for 3 months. We used a lead exposure of 0.075% lead acetate, comparably to exposure experiments reported in the literature. This level of exposure caused an increase in blood lead levels similar to that of modest poisoning (Zhu *et al.,* 2005; DeLuca *et al.,* 1982; Sun *et al.,* 2005). The controls were orally given sodium acetate. There were no significant differences in body weight at the beginning of the experiment. Body weight was recorded weekly.

### Determination of blood lead level

Immediately after decapitation of the mice, blood samples were collected in tubes pre-treated with 10 μl of heparin. The lead concentration in blood was determined by using graphite furnace atomic absorption spectroscopy (PE-700AA, Perkin-Elmer, USA). For determining lead in blood, the following temperature program was experimentally selected as optimum: dry at 110 °C for 30 s and at 130 °C for 30 s, ash at 800 °C for 20 s, atomize at 1600 °C for 5 s.

### DNA and RNA extraction

Total RNA from spleen tissue of lead exposed animals (n=6) and controls (n=6) was extracted using Trizol (Invitrogen, USA) according to the manufacturer's instructions. RNA quality of each sample was determined using Agilent 2100 Bioanalyzer (Agilent Technologies, SantaClara, CA). Qualified total RNA was purified using RNeasy Mini Kit (QIAGEN, Germany).

### Illumina microarray analysis

RNA was converted to labeled cRNA and hybridized to Illumina mouse WG-6_v2_Bead Chip. Microarray Hybridization, washing and detection were performed using the Illumina Gene Expression System K it according to the manufacturer's protocol. Arrays were scanned with an Illumina BeadArray Reader confocal scanner. Initial microarray gene expression data were obtained using the gene expression analysis module of Illumina BeadStudio Application.

### Real time RT-PCR

The differential expression of calmodulin related genes was validated by real-time RT-PCR (ABI 7300, USA) with SYBR green (Qiagen, Germany). Cycling parameters were as follows: 15 min at 95 °C, then 40 cycles of 94 °C for 15 s, 55–58 °C annealing temperature for 30 s, and extension for 30 s at 72 °C. The mRNA levels of genes were quantified by measuring the threshold cycle (Ct). Glyceraldehyde-3- phosphate dehydrogenase (GAPDH) was simultaneously assayed by real time RT-PCR as an endogenous invariant control. All primers used for PCR in this study are listed in Table 1.

**Table 1:** Primer list.

Primer (Target gene)	Direction	Sequence(5‘-3’)
CAMKK2	F	AGCAG CAACA GCCTG GACAT
	R	ATTCA GCTGC ACGCA GTCCT
CXCR4	F	TCCAC GCCAC CAACA GTCA
	R	AGGCG GTCAC AGATG TACCT GT
MAP2K6	F	GTCCA TTCAC CGTGA CCTTC TA
	R	GACGT CTCGA TGGAT AACGA ACA
PHKA2	F	CAGCC CTGCC ATCTC CATC
	R	CTCGC CTATT CATCT GTTTC ATC
MYLK	F	GATGA TCTAG TTAGG CTATT TGGA
	R	AATTA GACGA GTTGC TGGTG A
GAPDH	F	ACATG TTCCA GTATG ACTCC ACTCA
	R	TGAAG ACACC AGTAG ACTCC ACGA

#### Statistical analysis

Data entry was performed using SPSS 10.0. Results are presented as mean ± standard error (SE). A p-value of less than 0.05 was considered statistically significant.

#### Ethical considerations

This study was approved by the Academic Medical Center Animal Ethics Committee and complies with the guidelines for the care of experimental animals.

### Results

#### Assessment of lead levels

Blood lead levels were measured by atomic absorption spectrometry (PE-700AA). Lead concentrations of blood were 8.39±3.28 μg/dl in control mice and significantly higher with 28.08±9.43 μg/dl in lead-exposed mice (*p*<0.01, Table 2).

**Table 2:** Blood lead levels after lead exposure.

Group	N	Blood lead levels ($\bar{\text{x}}\pm \text{s}$, µg/dl)
Lead exposure	18	28.08±9.43
Control	18	8.39±3.28

Blood lead levels were significantly higher in the lead exposed than in the control group (*p*<0.01).

#### Microarray data analysis

There were 2216 differentially expressed genes with a false discovery rate (FDR) <0.05. Analysis of biological pathways, including Gene Onto logy (GO) and Kyoto Encyclopedia of Genes and Genomes (KEGG), indicated extreme overrepresentation of immune related categories. GO analysis of differentially expressed genes revealed that in biological processes the highly enriched categories included those related to metabolic processes, apoptosis, and macromolecule localization. As to molecular function, the highly enriched categories included those related to binding, catalytic activity and transferase activity, while in cellular component the highly enriched categories included those related to organelles, cytoplasm and nucleus. KEGG pathway analysis showed that 10 differentially expressed immune-related genes were involved in the pathway. By microarray analysis, we found five calmodulin-related genes (MAP2K6, CAMKK2, CXCR4, PHKA2, MYLK) that were differentially expressed in lead exposed compared with control mice. These included four genes with a higher and one with a lower expression level in lead exposed *vs.* control mice (*p*<0.05, Table 3).

**Table 3:** Differently expressed calmodulin-related genes in lead exposed and control mice by array analysis.

Gene	Gene ID	Description	Array Data (Fold change)	Diff Score
MAP2K6	26399	phosphokinase activity	7.21	24.56
CAMKK2	207565	calcium-mediated signaling	1.84	34.76
PHKA2	110094	calmodulin binding	1.76	18.07
MYLK	107589	calmodulin binding, calcium ion binding	1.43	17.43
CXCR4	12767	calcium-mediated signaling	0.24	–258.11

Microarray analysis showed that there were differences in gene expression between lead exposed and control mice. These included 4 genes with a higher and 1 with a lower expression level in lead exposed vs. control mice (*p*<0.05).

#### Confirmation of differential expression of calmodulin-related genes

**Figure 1:**
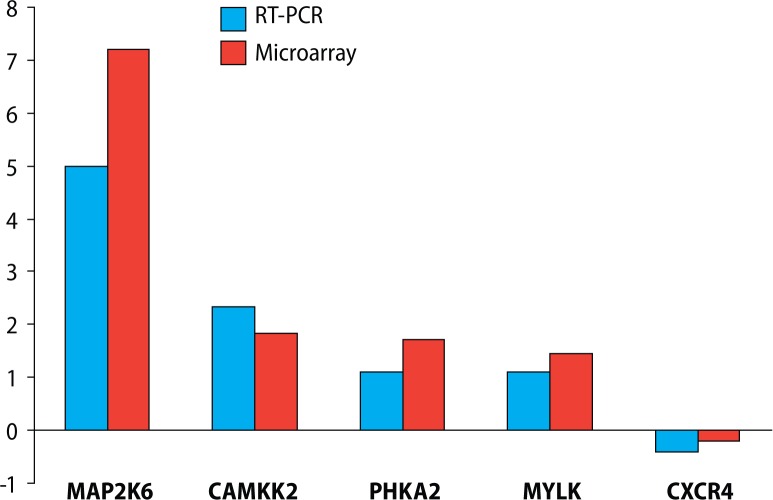
Histogram showing the expression values of selected 5 genes measured by microarray and real time PCR. The Y-axis shows the ratio (expressed as fold change) of gene expression between lead-exposed mice and control mice for each gene.

In order to verify the microarray data, five differentially expressed calmodulin-related genes (MAP2K6, CAMKK2, CXCR4, PHKA2 and MYLK) were selected and then subjected to real-time quantitative RT-PCR analysis. Expression levels of both MAP2K6 and CAMKK2 mRNAs were up-regulated in lead exposure, while CXCR4 was down-regulated. There were no significant differences in PHKA2 and MYLK expression between the lead exposed and control group. The results showed that expression of 3 genes exhibited change trends similar to those revealed by microarray data (Figure 1).

### Discussion

Lead is a widely dispersed and persistent environmental contaminant (Taylor *et al.,* 2014). It is toxic to many organ systems, including the immune system. Numerous studies support the hypothesis that lead is involved in altering cellular second messenger systems. Calmodulin, through its interaction with a large number of enzymes, is a primary mediator of cellular responses to Ca^2+^ fluxes (Taylor *et al.,* 2014). Calmodulin has four Ca^2+^ binding sites. Pb^2+^ binds not only to these but also to a “second class” of calmodulin binding sites, to which calcium does not bind. The binding of lead ions at these sites alters protein conformation (Simons, 1986). This may cause an altered effect on the activation of protein kinases. In a previous study, we reported that higher Pb^2+^ concentrations replaced produced by led to down-regulation of CaM content, but failure of lower Pb^2+^ exposure (Sun, 2012).

In this study, we focused on the expression of calmodulin-related genes. We discovered multiple genes which had not been previously implicated in lead exposure. We found 5 calmodulin-related genes (MAP2K6, CAMKK2, CXCR4, PHKA2, MYLK) that were differentially expressed in lead exposed compared with control animals, as shown by microarray analysis. These included 4 genes with a higher and 1 with a lower expression level in lead exposed vs. control mice (*p*<0.05, Table 3). In order to verify the microarray data, 5 of the differentially expressed calmodulin-related genes (MAP2K6, CAMKK2, CXCR4, PHKA2 and MYLK) were selected and then subjected to real-time quantitative RT-PCR analysis. Expression levels of both MAP2K6 and CAMKK2 mRNAs were upregulated, and CXCR4 was down-regulated in lead. There were no significant differences in PHKA2 and MYLK expression between lead exposed and control group. Thus 3 of the 5 genes were confirmed as being significantly different in lead exposure, while differential expression of 2 of the 5 genes was inferred from the microarray data but could not be confirmed by real-time PCR. Such disagreement is probably due to heterogeneity of the spleen tissues samples. MAP2K6 gene encodes a member of the dual specificity protein kinase family, which functions as a mitogen-activated protein (MAP) kinase. MAP kinases act as an integration point for multiple biochemical signals. This protein phosphorylates and activates p38 MAP kinase in response to inflammatory cytokines or environmental stress. As an essential component of p38 MAP kinase mediated signal transduction pathway, this gene is involved in many cellular processes such as stress induced cell cycle arrest, transcription activation and apoptosis (Gardner *et al.,* 2005; Mainiero *et al.,* 2003). CAMKK2, whose mRNA was significantly increased in lead exposure, is one of the most versatile of the CaMKs and will phosphorylate and activate CaMKI, CaMKIV, and AMP-activated protein kinase. CaMKK2 is involved in regulating many important physiological and pathophysiological processes, including energy balance, hematopoiesis, inflammation, and cancer. CaMKK2 expression leads to the activation of a kinase(s) downstream of CaMKK2, which in turn up-regulates cell cycle proteins. This could occur by direct phosphorylation of the cell cycle regulators, activation of their transcription or more indirectly, by regulation of anabolic metabolism (Racioppi *et al.,* 2012; Chen *et al.,* 2012). CXCR4, which showed decreased expression in lead exposure, is expressed on multiple cell types, including lymphocytes, hematopoietic stem cells, endothelial and epithelial cells, and cancer cells. Signal transduction pathways induce intracellular signaling through several divergent pathways initiating signals related to chemotaxis, increase in intracellular calcium, gene transcription, and cell survival and /or proliferation (Katsumoto *et al.,* 2011; Fernandis *et al.,* 2003).

### Conclusion

These preliminary data are significant in suggesting that lead plays a role in mediating the expression of calmodulin-related genes and that the mechanism of lead-induced toxicity is multifactorial. Further studies need to be conducted to ascertain the role of MAP2K6, CAMKK2 and CXCR4 signaling in lead exposure.
